# Risk of all-cause and cause-specific mortality associated with immune-mediated inflammatory diseases in Korea

**DOI:** 10.3389/fmed.2023.1185300

**Published:** 2023-06-20

**Authors:** Oh Chan Kwon, See Young Lee, Jaeyoung Chun, Kyungdo Han, Yuna Kim, Ryul Kim, Min-Chan Park, Jie-Hyun Kim, Young Hoon Youn, Hyojin Park

**Affiliations:** ^1^Division of Rheumatology, Department of Internal Medicine, Yonsei University College of Medicine, Seoul, Republic of Korea; ^2^Division of Gastroenterology, Department of Internal Medicine, Yonsei University College of Medicine, Seoul, Republic of Korea; ^3^Department of Statistics and Actuarial Science, Soongsil University, Seoul, Republic of Korea; ^4^Department of Neurology, Inha University Hospital, Incheon, Republic of Korea

**Keywords:** immune-mediated inflammatory diseases, risk, comorbidities, all-cause mortality, cause-specific mortality

## Abstract

**Objective:**

Immune-mediated inflammatory disease (IMID) is associated with an increased risk of mortality. It is unclear whether the higher mortality is attributable to the IMIDs themselves or to the higher prevalence of comorbidities in IMIDs. We aimed to investigate whether IMIDs *per se* confer a higher risk of mortality.

**Methods:**

From the Korean National Health Insurance Service-National Sample Cohort database, this population-based cohort study included 25,736 patients newly diagnosed with IMIDs between January 2007 and December 2017, and 128,680 individuals without IMIDs who were matched for age, sex, income, hypertension, type 2 diabetes, dyslipidemia, and the Charlson comorbidity index. All individuals were retrospectively observed through December 31, 2019. The outcomes included all-cause and cause-specific mortalities. Adjustments for age, sex, and comorbidities were performed using multivariable Cox proportional hazard regression analyses, and adjusted hazard ratios (aHRs) with 95% confidence intervals (CIs) for the outcomes were estimated.

**Results:**

The adjusted risk of all-cause mortality was significantly lower in patients with IMIDs than that in those without (aHR, 0.890; 95% CI, 0.841–0.942). Regarding cause-specific mortality, cancer-specific (aHR, 0.788; 95% CI, 0.712–0.872) and cardiovascular disease-specific (aHR, 0.798; 95% CI, 0.701–0.908) mortalities were the two causes of death that showed significantly lower risks in patients with IMIDs. A similar trend was observed when organ based IMIDs were analyzed separately (i.e., gut, joint, and skin IMIDs).

**Conclusion:**

After adjusting for comorbidities, IMIDs were associated with a lower risk of all-cause mortality compared to those without IMIDs. This was attributable to the lower risks of cancer-and cardiovascular disease-specific mortalities.

**Graphical Abstract:**
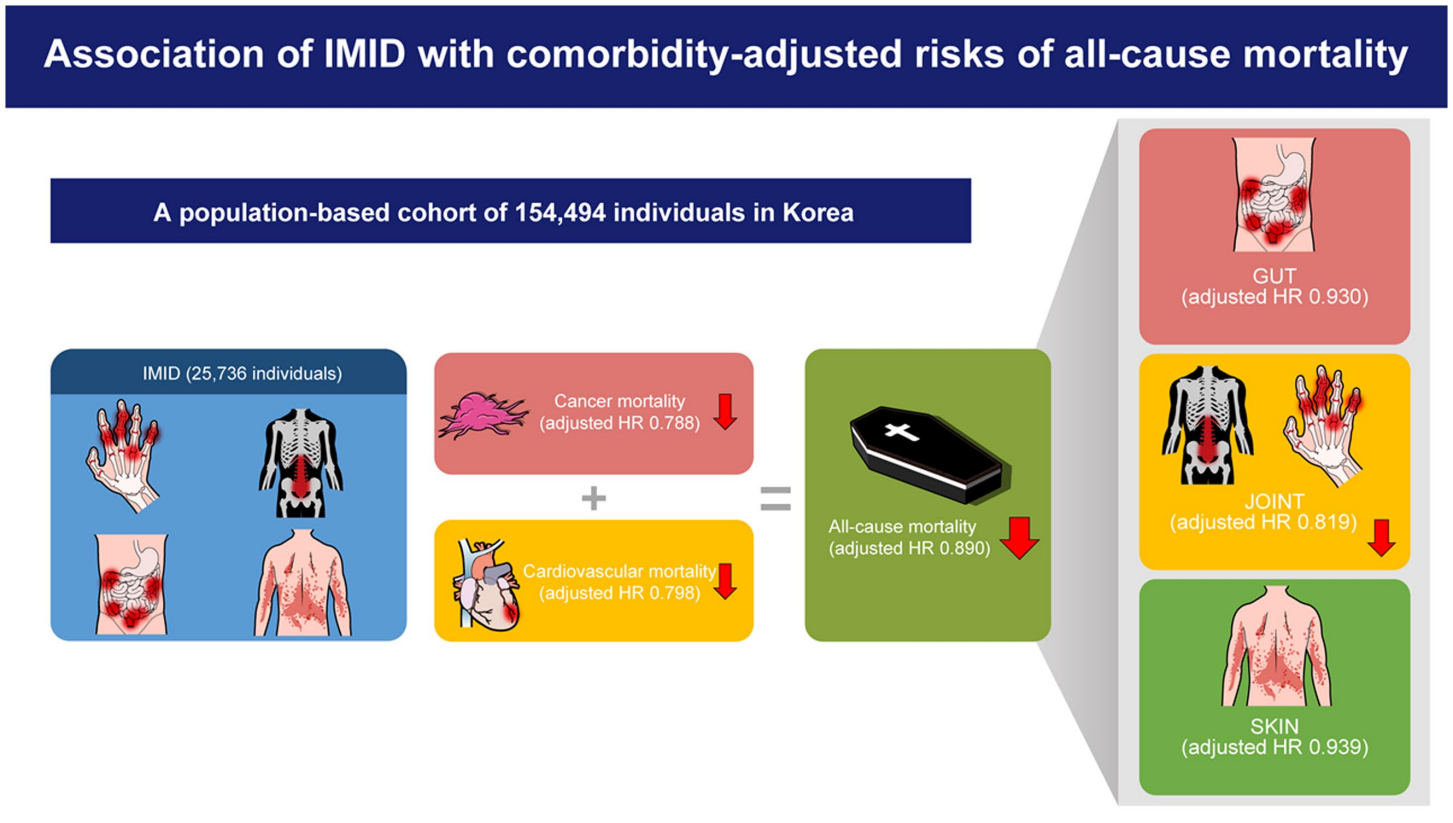
HR, hazard ratio; IMID, immune-mediated inflammatory disease.

## Introduction

Immune-mediated inflammatory diseases (IMIDs) are a heterogeneous group of diseases characterized by chronic inflammation and damage to various organs (1–3). These include Crohn’s disease (CD) and ulcerative colitis (UC), which affect the gut; rheumatoid arthritis (RA) and ankylosing spondylitis (AS), which affect the joints; and psoriasis (PsO), which affects the skin (4–20).

A higher mortality rate has been consistently reported in patients with RA, AS, and PsO than that among the general population (21–23). Patients with RA have a 1.4-to 2-fold high mortality rate (21); those with AS have a 1.8-fold high mortality rate (22); patients with PsO have a 1.2-fold high risk of mortality than the rates among the general population (23). Less consistent results have been reported regarding mortality in patients with inflammatory bowel disease (IBD) (24–26). A population-based study from Canada reported 1.5-fold and 1.2-fold high mortality in patients with CD and UC, respectively, compared with the rates in the general population (24). In contrast, studies from the United States (25) and South Korea (26) reported a higher mortality trend among patients with CD and lower mortality among patients with UC than among the general population.

Although there are several mortality studies on IMIDs, the results were mostly adjusted for age and sex but not for comorbidities such as hypertension, type 2 diabetes, dyslipidemia, and cardiovascular disease. Moreover, patients with IMIDs are more prone to comorbidities than the general population (3, 27–32). Therefore, comorbidities should be adjusted to evaluate whether IMIDs are associated with the risk of mortality among the general population. In this study, we conducted a nationwide population-based study to estimate the age-, sex-and comorbidity-adjusted risks of all-cause and cause-specific mortalities according to the presence of IMIDs.

## Patients and methods

### Data source

Data from the Korean National Health Insurance Service-National Sample Cohort (NHIS-NSC) database were used in this study. The NHIS provides comprehensive data that includes demographics, socioeconomic status, medical treatments and procedures, disease diagnoses according to the International Classification of Diseases – Tenth Revision (ICD-10), and rare intractable disease (RID) registration information (33). In the Korean RID system, a diagnosis is based on the uniform diagnostic criteria provided by the NHI and is carefully reviewed by the corresponding healthcare institution and the NHI before registration. The profile of the data source has been described previously (34). The NHIS-NSC is a large-scale, population-based cohort comprising a representative sample of approximately 2% of the general Korean population.

### Study population

**Figure 1:**
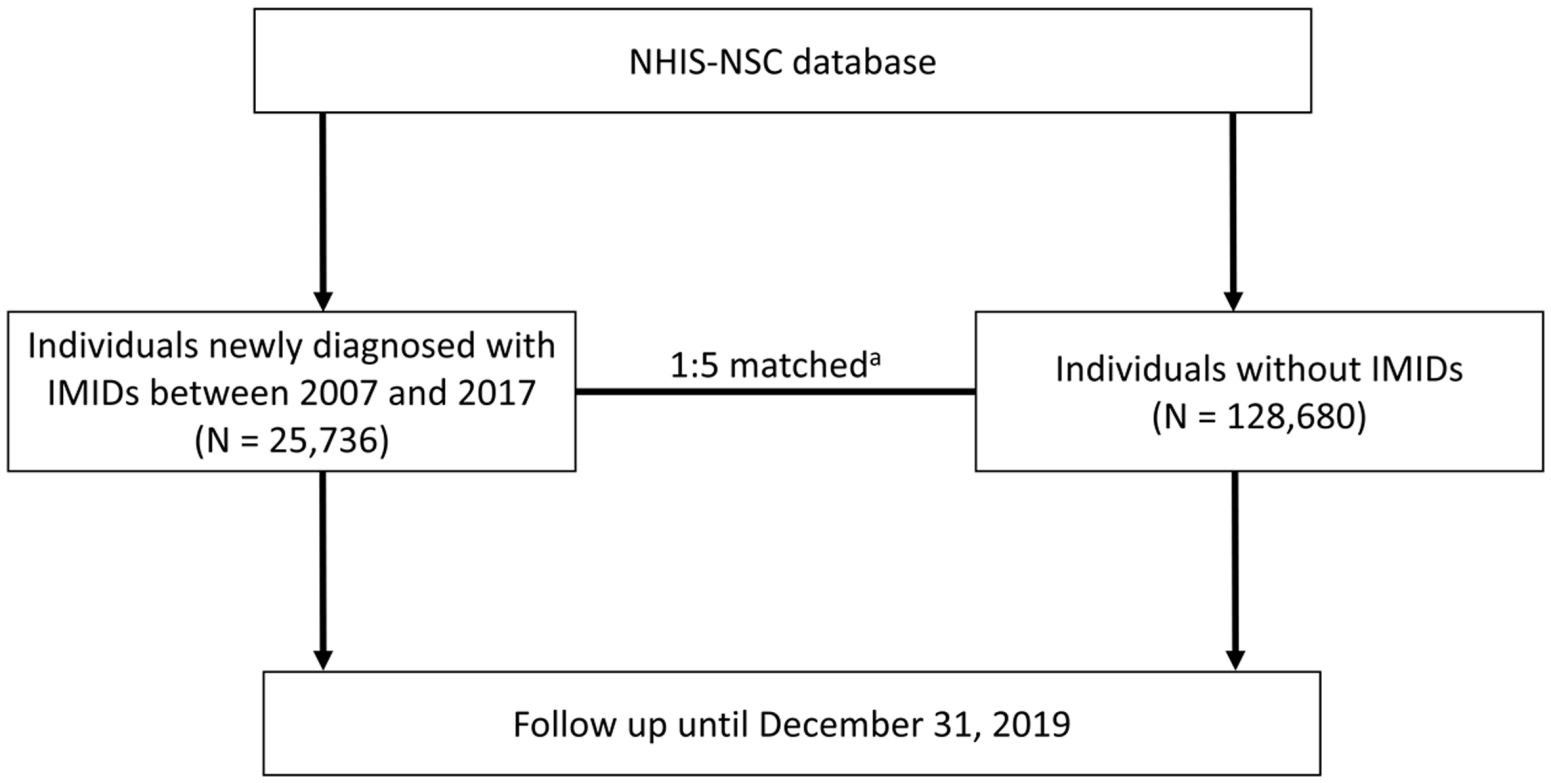
Flowchart of the study process. ^a^Matched by age, sex, income, hypertension, type 2 diabetes, dyslipidemia, and Charlson comorbidity index. NHIS-NSC, National Health Insurance Service-National Sample Cohort; IMIDs, immune-mediated inflammatory diseases.

From the NHIS-NSC database, we identified 25,736 patients who were newly diagnosed with IMIDs between January 1, 2007, and December 31, 2017 (IMID group). CD was defined as ICD-10 code K50 with RID code V130, UC was defined as ICD-10 code K51 with RID code V131, RA was defined as ICD-10 code M05 or M06 with prescriptions for disease-modifying anti-rheumatic drugs, AS was defined as ICD-10 code M45 with RID code V140, and PsO was defined as ICD-10 code L40 (35, 36). The control group (*N* = 128,680) was selected at a ratio of 1:5, matched for age, sex, income, hypertension, type 2 diabetes, dyslipidemia, and Charlson comorbidity index (CCI), respectively, with the IMID group. All individuals were followed up through December 31, 2019, for the occurrence of mortality (Figure 1).

### Mortality data

Data on mortality and causes of death were available for all individuals included in the study population. The cause of death was identified according to the Korean Standard Classification of Disease and Cause of Death, based on ICD-10 codes (37). We classified the causes of death into cancer (C00-97), cardiovascular disease (I00-99), infectious disease (J00-06, J09-18, J20-22, and J85-86), renal disease (N00-08 and N10-19), endocrine disease (E00-90), and digestive system disease (K00-93). Cancer-specific mortality was further divided into the top five cancers with the highest mortality in South Korea (lung, liver, stomach, pancreatic, and colorectal cancers) and others (38).

### Comorbidities

Presences of comorbidities were determined based on their existence prior to the diagnosis of IMIDs, and were defined as follows: hypertension was defined as ICD-10 codes I10–I13 and I15 with prescriptions for antihypertensive agents; type 2 diabetes was defined as ICD-10 codes E11–14, and at least one annual claim of a prescription of anti-diabetic medications; and dyslipidemia was defined as ICD-10 code E78 with prescriptions for lipid-lowering agents (35). CCI, which is a sum of weighted scores that are assigned to 17 comorbidities according to their relative risk of 1-year mortality, was calculated (39) and included as a covariate.

### Statistical analysis

Continuous variables were expressed as mean ± standard deviation, and categorical variables were expressed as numbers (%). Continuous variables were compared using an independent Student’s *t*-test, and categorical variables were compared using the *χ*^2^ test. All-cause and cause-specific mortality rates were expressed as the number of deaths per 1,000 person-years. All-cause and cause-specific standardized mortality ratio (SMR) were estimated using 2010 mortality statistics in Korea. Cause-specific hazard models based on multivariable Cox proportional hazard models adjusted for age, sex, income, hypertension, type 2 diabetes, dyslipidemia, and CCI were used to estimate the hazard ratios (HRs) and 95% confidence intervals (CI) for all-cause mortality and cause-specific mortality, respectively. In cause-specific hazard models, the competing risk events are treated as censored observations (40). This method is advantageous in that it directly quantifies the HRs among individuals who are actually at risk of developing the event of interest (41, 42). We first estimated the HRs and 95% CIs of all IMIDs as a whole compared with the controls and then estimated the HRs and 95% CIs of each organ-based IMID (gut IMIDs [CD or UC], joint IMIDs [RA or AS], and skin IMID [PsO]) compared with the controls. Subgroup analyses were performed according to age and sex. All *p* values were two sided, and a *p* value <0.05 was considered statistically significant. All the statistical analyses were performed using SAS version 9.4 (SAS Institute, Cary, NC, USA).

## Results

### Baseline characteristics

The baseline characteristics of the 25,764 patients with IMIDs and a comparison with those of the 1:5 matched control group (128,820) are shown in Table 1. The mean age of the patients with IMIDs was 48.5 ± 18.8 years, and 11,884 (46.1%) patients were male. One third of the patients (8,885; 34.5%) had a low income (lower 25 percentile). The proportion of patients with hypertension, type 2 diabetes, and dyslipidemia was 25.1, 9.7, and 16.3%, respectively. The mean CCI was 1.99 ± 1.99. The characteristics were well balanced through the matching process between the IMID and control groups, with absolute standardized differences below 0.1 for all covariates.

**Table 1:** Baseline characteristics of the study population.

	IMID group	Control group	*P* value	ASD
Age, years, mean ± SD	48.51 ± 18.75	48.68 ± 18.7	0.175	0.009241
20–39 years, *n* (%)	8,072 (31.33)	39,778 (30.88)	0.085	
40–59 years, *n* (%)	9,995 (38.79)	49,982 (38.8)		
60–79 years, *n* (%)	6,869 (26.66)	35,146 (27.28)		
≥80 years, *n* (%)	828 (3.21)	3,914 (3.04)		
Male sex, *n* (%)	11,884 (46.13)	60,101 (46.66)	0.121	0.010601
Low income, *n* (%)	8,885 (34.49)	44,039 (34.19)	0.355	0.006311
Hypertension, *n* (%)	6,472 (25.12)	32,493 (25.22)	0.728	0.002379
Type 2 diabetes, *n* (%)	2,493 (9.68)	12,012 (9.32)	0.077	0.011993
Dyslipidemia, *n* (%)	4,194 (16.28)	20,747 (16.11)	0.491	0.004699
CCI, mean ± SD	1.99 ± 1.99	1.98 ± 1.99	0.498	0.004621

ASD, absolute standardized difference; CCI, Charlson comorbidity index; IMID, immune-mediated inflammatory disease; SD, standard deviation.

### Mortality rate and risk of mortality according to the presence of IMIDs

The all-cause mortality rates of the IMID and control group were 10.43 and 12.13 per 1,000 person-years, respectively. The all-cause and cause-specific SMR were higher in patients with IMIDs than in unmatched general population (all-cause SMR, 1.404; cancer-specific SMR, 1.336; cardiovascular disease-specific SMR, 1.225; infectious disease-specific SMR, 2.127; renal disease-specific SMR, 2.054; endocrine disease-specific SMR, 1.678; and digestive system disease-specific SMR, 1.337). However, the results of the multivariable Cox model summarized in Table 2 revealed that, compared with matched controls, patients with IMIDs had a significantly reduced risk of all-cause mortality (adjusted hazard ratio [aHR], 0.890; 95% CI, 0.841–0.942). With regard to cause-specific mortality, patients with IMIDs had a significantly lower risk of cancer-specific mortality (aHR, 0.788; 95% CI, 0.712–0.872) and cardiovascular disease-specific mortality (aHR, 0.798; 95% CI, 0.701–0.908) than that in controls. In contrast, the risks of infectious disease-specific, renal disease-specific, endocrine disease-specific, and digestive system disease-specific mortality did not show significant differences between the IMID and control groups. When cancer-specific mortality was further classified according to the specific cancer type, patients with IMIDs had a significantly reduced risk of liver cancer-specific (aHR, 0.584; 95% CI, 0.436–0.782), stomach cancer-specific (aHR, 0.468; 95% CI, 0.324–0.676), and colorectal cancer-specific mortality (aHR, 0.540; 95% CI, 0.383–0.761), but not lung cancer-specific, pancreatic cancer-specific, or other cancer-specific mortalities.

**Table 2:** Mortality rate and hazard ratios for death according to the presence of IMIDs.

Cause of death	*N*	Death	MR (/1,000 person-years)	SMR^a^ (95% CI)	Unadjusted HR (95% CI)	Adjusted HR^b^ (95% CI)
All-cause						
Control	128,820	9,890	12.1253	1 (Ref.)	1 (Ref.)	1 (Ref.)
IMID	25,764	1789	10.4257	1.404 (1.339,1.469)	0.883 (0.835,0.933)	0.890 (0.841,0.942)
Cancer						
Control	128,820	3,227	3.95636	1 (Ref.)	1 (Ref.)	1 (Ref.)
IMID	25,764	528	3.07701	1.336 (1.222,1.450)	0.764 (0.692,0.844)	0.788 (0.712,0.872)
Lung cancer^c^						
Control	128,820	745	0.91338	1 (Ref.)	1 (Ref.)	1 (Ref.)
IMID	25,764	156	0.90912	1.741 (1.468,2.014)	1.028 (0.851,1.242)	1.056 (0.870,1.282)
Liver cancer^c^						
Control	128,820	509	0.62404	1 (Ref.)	1 (Ref.)	1 (Ref.)
IMID	25,764	59	0.34383	0.987 (0.745,1.238)	0.558 (0.420,0.743)	0.584 (0.436,0.782)
Stomach cancer^c^						
Control	128,820	368	0.45118	1 (Ref.)	1 (Ref.)	1 (Ref.)
IMID	25,764	38	0.22145	0.706 (0.481,0.930)	0.461 (0.323,0.656)	0.468 (0.324,0.676)
Pancreatic cancer^c^						
Control	128,820	201	0.24643	1 (Ref.)	1 (Ref.)	1 (Ref.)
IMID	25,764	32	0.18649	1.340 (0.876,1.804)	0.650 (0.430,0.984)	0.675 (0.443,1.028)
Colorectal cancer^c^						
Control	128,820	378	0.46344	1 (Ref.)	1 (Ref.)	1 (Ref.)
IMID	25,764	45	0.26224	1.073 (0.759,1.386)	0.490 (0.350,0.686)	0.540 (0.383,0.761)
Other cancers^d^						
Control	128,820	1,026	1.2579	1 (Ref.)	1 (Ref.)	1 (Ref.)
IMID	25,764	198	1.15388	1.571 (1.352,1.790)	0.935 (0.793,1.103)	0.933 (0.790,1.103)
Cardiovascular disease						
Control	128,820	2090	2.56238	1 (Ref.)	1 (Ref.)	1 (Ref.)
IMID	25,764	345	2.01054	1.225 (1.096,1.355)	0.810 (0.715,0.917)	0.798 (0.701,0.908)
Infectious disease						
Control	128,820	1,050	1.28732	1 (Ref.)	1 (Ref.)	1 (Ref.)
IMID	25,764	198	1.15388	2.127 (1.830,2.423)	0.976 (0.821,1.16)	0.978 (0.818,1.169)
Renal disease						
Control	128,820	294	0.36045	1 (Ref.)	1 (Ref.)	1 (Ref.)
IMID	25,764	52	0.30304	2.054 (1.496,2.613)	0.865 (0.623,1.202)	0.909 (0.644,1.284)
Endocrine disease						
Control	128,820	433	0.53087	1 (Ref.)	1 (Ref.)	1 (Ref.)
IMID	25,764	98	0.57111	1.678 (1.346,2.010)	1.131 (0.887,1.442)	1.066 (0.824,1.38)
Digestive system disease						
Control	128,820	452	0.55416	1 (Ref.)	1 (Ref.)	1 (Ref.)
IMID	25,764	73	0.42542	1.337 (1.052,1.678)	0.793 (0.604,1.039)	0.814 (0.618,1.071)

^a^SMR was calculated using 2010 mortality statistics in Korea. ^b^Adjusted for age, sex, income, hypertension, type 2 diabetes, dyslipidemia, and CCI.^c^The top 5 cancers of highest mortality in Korea. ^d^Cancers other than the top 5 cancers of highest mortality in Korea. CI, confidence interval; HR, hazard ratio; IMID, immune-mediated inflammatory disease; MR, mortality rate; SMR, standardized mortality ratio.

As we included patients newly diagnosed with IMIDs between 2007 and 2017, the disease duration of IMIDs may vary among patients at the end of follow up (2019). To address the variable disease duration among patients, we stratified the patients into those who were newly diagnosed with IMIDs in 2012–2017, and those who were newly diagnosed with IMIDs in 2007–2011, and performed Cox models in each group. The results are shown in Tables 3, 4. In patients who were newly diagnosed with IMIDs in 2012–2017, similar results were observed as in the total study population (reduced risk of all-cause mortality [aHR, 0.765; 95% CI, 0.701–0.834], cancer-specific mortality [aHR, 0.693; 95% CI, 0.595–0.806], and cardiovascular disease-specific mortality [aHR, 0.646; 95% CI, 0.527–0.792]) (Table 3). On the other hand, in patients who were newly diagnosed with IMIDs in 2007–2011, the reduced risk of all-cause mortality (aHR, 1.007; 95% CI, 0.934–1.086), cancer-specific mortality (aHR, 0.882; 95% CI, 0.768–1.012), and cardiovascular disease-specific mortality (aHR, 0.934; 95% CI, 0.789–1.105) was not observed (Table 4).

**Table 3:** Mortality rate and hazard ratios for death according to the presence of IMIDs (newly diagnosed in 2012–2017).

Cause of death	*N*	Death	MR (/1,000 person-years)	Unadjusted HR (95% CI)	Adjusted HR^a^ (95% CI)
All-cause					
Control	76,920	6,063	12.5836	1 (Ref.)	1 (Ref.)
IMID	15,384	672	9.8538	0.778 (0.714,0.846)	0.765 (0.701,0.834)
Cancer					
Control	76,920	1979	4.10737	1 (Ref.)	1 (Ref.)
IMID	15,384	213	3.1233	0.68 (0.586,0.789)	0.693 (0.595,0.806)
Lung cancer^b^					
Control	76,920	458	0.95057	1 (Ref.)	1 (Ref.)
IMID	15,384	65	0.95312	0.925 (0.701,1.22)	0.96 (0.721,1.277)
Liver cancer^b^					
Control	76,920	295	0.61227	1 (Ref.)	1 (Ref.)
IMID	15,384	28	0.41057	0.608 (0.407,0.907)	0.63 (0.418,0.95)
Stomach cancer^b^					
Control	76,920	224	0.46491	1 (Ref.)	1 (Ref.)
IMID	15,384	8	0.11731	0.231 (0.113,0.472)	0.252 (0.122,0.519)
Pancreatic cancer^b^					
Control	76,920	124	0.25736	1 (Ref.)	1 (Ref.)
IMID	15,384	9	0.13197	0.429 (0.213,0.863)	0.435 (0.213,0.885)
Colorectal cancer^b^					
Control	76,920	235	0.48774	1 (Ref.)	1 (Ref.)
IMID	15,384	19	0.2786	0.462 (0.285,0.747)	0.509 (0.311,0.833)
Other cancers^c^					
Control	76,920	643	1.33453	1 (Ref.)	1 (Ref.)
IMID	15,384	84	1.23172	0.848 (0.665,1.082)	0.834 (0.652,1.067)
Cardiovascular disease					
Control	76,920	1,289	2.67529	1 (Ref.)	1 (Ref.)
IMID	15,384	125	1.83292	0. 701 (0.577,0.852)	0.646 (0.527,0.792)
Infectious disease					
Control	76,920	650	1.34906	1 (Ref.)	1 (Ref.)
IMID	15,384	78	1.14374	0.952 (0.738,1.227)	0.946 (0.726,1.234)
Renal disease					
Control	76,920	185	0.38396	1 (Ref.)	1 (Ref.)
IMID	15,384	19	0.2786	0.718 (0.436,1.182)	0.779 (0.466,1.303)
Endocrine disease					
Control	76,920	259	0.53755	1 (Ref.)	1 (Ref.)
IMID	15,384	32	0.46923	0.921 (0.622,1.365)	0.801 (0.525,1.22)
Digestive system disease					
Control	76,920	286	0.59359	1 (Ref.)	1 (Ref.)
IMID	15,384	33	0.48389	0.791 (0.537,1.163)	0.814 (0.549,1.206)

^a^Adjusted for age, sex, income, hypertension, type 2 diabetes, dyslipidemia, and CCI. ^b^The top 5 cancers of highest mortality in Korea. ^c^Cancers other than the top 5 cancers of highest mortality in Korea. CI, confidence interval; HR, hazard ratio; IMID, immune-mediated inflammatory disease; MR, mortality rate.

**Table 4:** Mortality rate and hazard ratios for death according to the presence of IMIDs (newly diagnosed in 2007–2011).

Cause of death	*N*	Death	MR (/1,000 person-years)	Unadjusted HR (95% CI)	Adjusted HR^a^ (95% CI)
All-cause					
Control	51,900	3,827	11.4639	1 (Ref.)	1 (Ref.)
IMID	10,380	1,117	10.8029	0.978 (0.908,1.053)	1.007 (0.934,1.086)
Cancer					
Control	51,900	1,248	3.73843	1 (Ref.)	1 (Ref.)
IMID	10,380	315	3.04647	0.847 (0.74,0.969)	0.882 (0.768,1.012)
Lung cancer^b^					
Control	51,900	287	0.85972	1 (Ref.)	1 (Ref.)
IMID	10,380	91	0.88009	1.134 (0.874,1.472)	1.166 (0.891,1.525)
Liver cancer^b^					
Control	51,900	214	0.64104	1 (Ref.)	1 (Ref.)
IMID	10,380	31	0.29981	0.514 (0.343,0.772)	0.536 (0.351,0.819)
Stomach cancer^b^					
Control	51,900	144	0.43136	1 (Ref.)	1 (Ref.)
IMID	10,380	30	0.29014	0.66 (0.433,1.004)	0.672 (0.431,1.048)
Pancreatic cancer^b^					
Control	51,900	77	0.23066	1 (Ref.)	1 (Ref.)
IMID	10,380	23	0.22244	0.876 (0.517,1.485)	0.964 (0.56,1.657)
Colorectal cancer^b^					
Control	51,900	143	0.42836	1 (Ref.)	1 (Ref.)
IMID	10,380	26	0.25146	0.52 (0.324,0.834)	0.578 (0.358,0.933)
Other cancers^c^					
Control	51,900	383	1.14729	1 (Ref.)	1 (Ref.)
IMID	10,380	114	1.10253	1.023 (0.815,1.283)	1.019 (0.809,1.283)
Cardiovascular disease					
Control	51,900	801	2.39942	1 (Ref.)	1 (Ref.)
IMID	10,380	220	2.1277	0.904 (0.767,1.064)	0.934 (0.789,1.105)
Infectious disease					
Control	51,900	400	1.19821	1 (Ref.)	1 (Ref.)
IMID	10,380	120	1.16056	0.997 (0.788,1.261)	1.018 (0.8,1.296)
Renal disease					
Control	51,900	109	0.32651	1 (Ref.)	1 (Ref.)
IMID	10,380	33	0.31915	1.017 (0.654,1.583)	1.039 (0.645,1.676)
Endocrine disease					
Control	51,900	174	0.52122	1 (Ref.)	1 (Ref.)
IMID	10,380	66	0.63831	1.301 (0.953,1.777)	1.285 (0.923,1.789)
Digestive system disease					
Control	51,900	166	0.49726	1 (Ref.)	1 (Ref.)
IMID	10,380	40	0.38685	0.794 (0.543,1.162)	0.832 (0.564,1.228)

^a^Adjusted for age, sex, income, hypertension, type 2 diabetes, dyslipidemia, and CCI. ^b^The top 5 cancers of highest mortality in Korea. ^c^Cancers other than the top 5 cancers of highest mortality in Korea. CI, confidence interval; HR, hazard ratio; IMID, immune-mediated inflammatory disease; MR, mortality rate.

### Mortality rate and risk of mortality according to the presence of organ-based IMIDs

The all-cause mortality rates of patients with gut, joint, and skin IMIDs were 5.39, 11.17, and 10.32 per 1,000 person-years, respectively. In the multivariable Cox model (Table 5), patients with joint IMIDs had a significantly lower risk of all-cause mortality (aHR, 0.819; 95% CI, 0.747–0.898) than the controls. Regarding cause-specific mortality, patients with joint IMIDs had a significantly lower risk of cancer-specific mortality (aHR, 0.512; 95% CI, 0.428–0.612), those with skin IMID had a significantly low risk of cardiovascular disease-specific mortality (aHR, 0.790; 95% CI, 0.668–0.935), those with joint IMIDs had a significantly high risk of infectious disease-specific mortality (aHR, 1.397; 95% CI, 1.059–1.843), those with skin IMID had a significantly low risk of infectious disease-specific mortality (aHR, 0.778; 95% CI, 0.613–0.988), and those with gut IMIDs had a significantly higher risk of disease-specific mortality in the digestive system (aHR, 5.407; 95% CI, 1.481–19.740) than the controls. In addition, the risk of renal disease-specific mortality and endocrine disease-specific mortality did not show significant differences between patients with organ-based IMIDs and controls.

**Table 5:** Mortality rate and hazard ratios for death according to the presence of organ-based IMIDs.

Cause of death	*N*	Death	MR (/1,000 person-years)	Adjusted HR^a^ (95% CI)
All-cause				
Control	128,820	9,890	12.1253	1 (Ref.)
Gut IMIDs	1,025	37	5.3895	0.93 (0.635,1.364)
Joint IMIDs	8,906	676	11.1726	0.819 (0.747,0.898)
Skin IMID	15,833	1,076	10.3238	0.939 (0.873,1.01)
Cancer				
Control	128,820	3,227	3.95636	1 (Ref.)
Gut IMIDs	1,025	9	1.31097	0.73 (0.357,1.494)
Joint IMIDs	8,906	159	2.62788	0.512 (0.428,0.612)
Skin IMID	15,833	360	3.45406	1.031 (0.908,1.17)
Cardiovascular disease				
Control	128,820	2090	2.56238	1 (Ref.)
Gut IMIDs	1,025	2	0.29133	0.268 (0.063,1.15)
Joint IMIDs	8,906	133	2.19816	0.837 (0.682,1.028)
Skin IMID	15,833	210	2.01487	0.79 (0.668,0.935)
Infectious disease				
Control	128,820	1,050	1.28732	1 (Ref.)
Gut IMIDs	1,025	4	0.58265	0.812 (0.206,3.207)
Joint IMIDs	8,906	93	1.53706	1.397 (1.059,1.843)
Skin IMID	15,833	101	0.96906	0.778 (0.613,0.988)
Renal disease				
Control	128,820	294	0.36045	1 (Ref.)
Gut IMIDs	1,025	0	0	-
Joint IMIDs	8,906	17	0.28097	0.603 (0.334,1.088)
Skin IMID	15,833	35	0.33581	1.192 (0.774,1.835)
Endocrine disease				
Control	128,820	433	0.53087	1 (Ref.)
Gut IMIDs	1,025	3	0.43699	0.606 (0.119,3.08)
Joint IMIDs	8,906	32	0.52888	0.829 (0.534,1.287)
Skin IMID	15,833	63	0.60446	1.276 (0.92,1.769)
Digestive system disease				
Control	128,820	452	0.55416	1 (Ref.)
Gut IMIDs	1,025	6	0.87398	5.407 (1.481,19.74)
Joint IMIDs	8,906	26	0.42972	0.659 (0.416,1.045)
Skin IMID	15,833	41	0.39338	0.815 (0.566,1.175)

^a^Adjusted for age, sex, income, hypertension, type 2 diabetes, dyslipidemia, and CCI. CI, confidence interval; HR, hazard ratio; IMID, immune-mediated inflammatory disease; MR, mortality rate.

When organ-based IMIDs were further subdivided into individual IMIDs (Table 6), the trends were similar between CD and UC (i.e., gut IMIDs) and between RA and AS (i.e., joint IMIDs), except for the risk of all-cause mortality in CD and UC in which CD showed a trend of high risk of mortality (aHR, 2.160; 95% CI, 0.995–4.687), whereas UC showed a trend of low risk of mortality (aHR, 0.747; 95% CI, 0.480–1.165).

**Table 6:** Mortality rate and hazard ratios for death according to the presence of each IMID.

Cause of death	*N*	Death	MR (/1,000 person-years)	Adjusted HR^a^ (95% CI)
All-cause				
Control	128,820	9,890	12.1253	1 (Ref.)
CD	347	10	4.229	2.16 (0.995,4.687)
UC	678	27	5.9993	0.747 (0.48,1.165)
RA	8,306	636	11.2624	0.815 (0.742,0.896)
AS	600	40	9.9159	0.893 (0.605,1.318)
PsO	15,833	1,076	10.3238	0.939 (0.873,1.01)
Cancer				
Control	128,820	3,227	3.95636	1 (Ref.)
CD	347	1	0.4229	0.483 (0.062,3.789)
UC	678	8	1.77756	0.783 (0.364,1.683)
RA	8,306	153	2.70935	0.519 (0.433,0.622)
AS	600	6	1.48738	0.344 (0.12,0.982)
PsO	15,833	360	3.45406	1.031 (0.908,1.17)
Cardiovascular disease				
Control	128,820	2090	2.56238	1 (Ref.)
CD	347	0	0	-
UC	678	2	0.44439	0.269 (0.063,1.151)
RA	8,306	122	2.1604	0.824 (0.666,1.02)
AS	600	11	2.72686	1.026 (0.484,2.177)
PsO	15,833	210	2.01487	0.79 (0.668,0.935)
Infectious disease				
Control	128,820	1,050	1.28732	1 (Ref.)
CD	347	0	0	-
UC	678	4	0.88878	0.873 (0.216,3.528)
RA	8,306	87	1.54061	1.391 (1.045,1.851)
AS	600	6	1.48738	1.495 (0.489,4.572)
PsO	15,833	101	0.96906	0.778 (0.613,0.988)
Renal disease				
Control	128,820	294	0.36045	1 (Ref.)
CD	347	0	0	-
UC	678	0	0	-
RA	8,306	16	0.28333	0.558 (0.304,1.027)
AS	600	1	0.2479	5.906 (0.332,105.064)
PsO	15,833	35	0.33581	1.192 (0.774,1.835)
Endocrine disease				
Control	128,820	433	0.53087	1 (Ref.)
CD	347	0	0	-
UC	678	3	0.66659	0.796 (0.144,4.391)
RA	8,306	30	0.53125	0.769 (0.488,1.211)
AS	600	2	0.49579	5.63 (0.745,42.539)
PsO	15,833	63	0.60446	1.276 (0.92,1.769)
Digestive system disease				
Control	128,820	452	0.55416	1 (Ref.)
CD	347	2	0.8458	7.717 (0.682,87.367)
UC	678	4	0.88878	4.65 (0.997,21.698)
RA	8,306	26	0.46041	0.731 (0.459,1.166)
AS	600	0	0	-
PsO	15,833	41	0.39338	0.815 (0.566,1.175)

^a^Adjusted for age, sex, income, hypertension, type 2 diabetes, dyslipidemia, and CCI. AS, ankylosing spondylitis; CD, Crohn’s disease; CI, confidence interval; HR, hazard ratio; IMID, immune-mediated inflammatory disease; MR, mortality rate; PsO, psoriasis; RA, srheumatoid arthritis; UC, ulcerative colitis.

### Subgroup analysis

**Figure 2:**
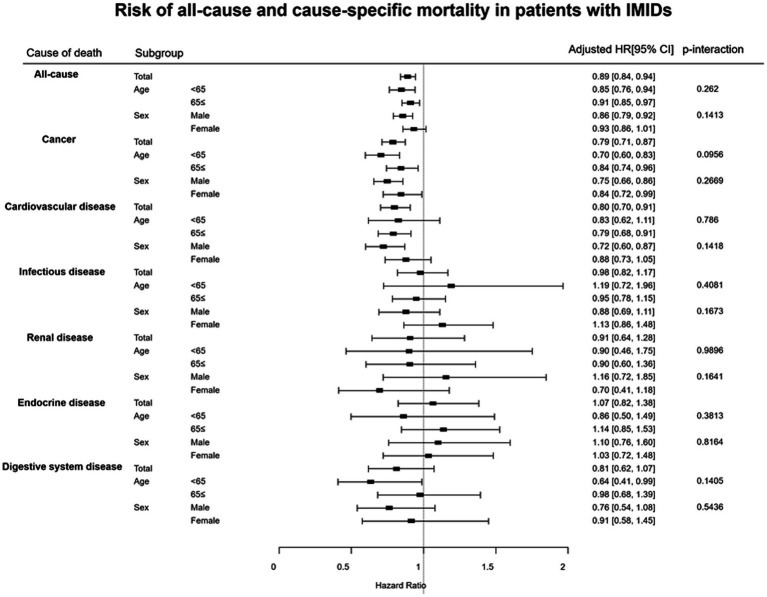
Subgroup analyses results for the risks of all-cause and cause-specific mortality in patients with IMIDs. CI, confidence interval; HR, hazard ratio; IMIDs, immune-mediated inflammatory diseases.

The results of subgroup analyses for the risk of all-cause mortality and cause-specific mortality are shown in Figure 2. There were no significant differences in the aHRs for all-cause mortality and cause-specific mortality between the subgroups of patients divided by age (<65 years vs. ≥ 65 years) and sex (male vs. female).

## Discussion

In this population-based cohort study in which patients with IMIDs and controls were matched for age, sex, and comorbidities, we observed that the presence of IMIDs was not associated with a high risk of all-cause mortality and cause-specific mortality. Rather, the presence of IMIDs was associated with a significantly low risk of all-cause, cancer-, and cardiovascular disease-specific mortality. Our findings provide important information that IMIDs themselves do not confer an additional risk of mortality when the influence of other comorbidities is minimized. To our best knowledge, this is the first study to compare mortality risk between patients with and without IMIDs after rigorous adjustment for comorbidities using matching and multivariable analyses.

In contrast to previous studies that compared mortality between patients with IMIDs and controls and reported a high risk of all-cause mortality in patients with IMIDs (21–24), we observed a low risk of all-cause mortality (aHR, 0.815) in patients with IMIDs that that in controls. However, it is important to note that the risk of all-cause mortality had not been fully adjusted for comorbidities in previous studies (21–24); therefore, the association between IMIDs and the risk of all-cause mortality could not be clearly stated. The higher risk of all-cause mortality in patients with IMIDs observed in previous studies could potentially be attributable to the differences in comorbidities between those with and without IMIDs. Indeed, patients with IMIDs have comorbidities more commonly than the general population (27–30). This study, in which the risk of mortality was adjusted for age, sex, and comorbidities, suggests that IMIDs *per se* confer a low risk of all-cause mortality. Meanwhile, the all-cause and cause-specific SMR were > 1 in our study, which means that when the influence of comorbidities is not adjusted, patients with IMIDs have higher mortality than the general population. Taken together, these emphasize that comorbidities are the main mediators of increased mortality in patients with IMIDs, whereas IMIDs themselves are not.

When patients were stratified into those who were newly diagnosed with IMIDs in 2012–2017 and those who were newly diagnosed with IMIDs in 2007–2011, and analyzed separately, the reduced risk of all-cause mortality, cancer-specific mortality, and cardiovascular disease-specific mortality was attenuated in the latter group (i.e., those who have longer disease duration at the end of follow up) and lost statistical significance. As the disease duration of IMIDs extends, more patients would likely develop comorbidities related to increased mortality, which might lead to the attenuation of mortality risk reduction that was observed in those with shorter disease duration. Although different results were observed based on the disease duration, it is important to note that IMIDs *per se* were not associated with additional risk of mortality following the adjustment for comorbidities at diagnosis, regardless of disease duration. Although direct statistical comparison of demographics and follow-up period cannot be made between our study and previous studies that reported higher mortality in patients with IMIDs (24, 43–48), there are some numerical differences to be noted (Supplementary Table S1). Patients with CD in our study were younger (mean age 29.07 years), more commonly male (64.27%), and followed for a longer period (mean 6.81 years) than those in previous studies [age, mean 42.8 years (43) and 72.13% of the patients older than 60 years (24); proportion of male, 41.3% (43) and 43.1% (24); and follow-up period, mean 3.62 years (43) and mean 4.67 years (24)]. Patients with UC in our study were younger (mean age 43.58 years), more commonly male (61.8%), and followed for a longer period (mean 6.64 years) than those in previous studies [age, mean 48.59 years (43) and 81.89% of the patients older than 60 years (24); proportion of male, 51.1% (43) and 56.7% (24); and follow-up period, mean 3.75 years (43) and mean 4.38 years (24)]. Patients with PsO in our study were younger (mean age 46.6 years), more commonly male (54.76%), and followed for a longer period (mean 6.58 years) than those in previous study (age, mean 52.19 years; proportion of male, 48.57%; and follow-up period, mean 3.43 years) (44). Patients with AS in our study were younger (mean age 41.52 years) and more commonly male (73.33%) than those in previous studies [age, mean 49.3 years (45) and mean 48.5 years (46); proportion of male, 65.5% (45) and 57.0% (46)]. The follow-up period (mean 6.72 years) of patients with AS in our study was similar to one study (mean 6.05 years) (45), but shorter than another study (mean 8.0 years) (46). Patients with RA in our study were younger (mean age 53.86 years) than those in previous studies [age, mean 58.5 years (47) and 58.0 years (48)]. The proportion of male (25.55%) in our study was similar to previous studies [20.1% (47) and 26.9% (48)]. The follow-up period (mean 6.8 years) of patients with RA in our study was longer than one study (mean 5 years) (47), but shorter than another study (mean 14.2 years) (48). Nonetheless, when the effect of comorbidities was not adjusted, we similarly observed higher mortality (i.e., SMR > 1) in patients with IMIDs as in the previous studies (24, 43–48).

Interestingly, regarding cause-specific mortality, a lower risk of mortality was observed in cancer-specific mortality (aHR, 0.788; the aHR was lowest for stomach cancer-specific mortality [0.468], followed by colorectal cancer [0.540] and liver cancer [0.584]) and cardiovascular disease-specific mortality (aHR, 0.798), but not for other cause-specific mortalities. Notably, cancer and cardiovascular disease are the two causes of death in which early detection is especially important for reducing mortality (49–53). Moreover, as the patients with IMIDs regularly visit the hospital for the management of their diseases, there is a higher chance of early detection of other serious diseases apart from the IMIDs. Hence, earlier detection of serious diseases, particularly cancer or cardiovascular disease, in patients with IMIDs through regular hospital visits is a possible explanation for the lower risk of cancer-specific and cardiovascular disease-specific mortality and, ultimately, the all-cause mortality observed in this group of patients.

Chronic inflammation, the hallmark of IMIDs, plays a major role in the pathogenesis of endothelial dysfunction, which leads to accelerated atherosclerosis (54). In particular, tumor necrosis factor (TNF)-α, the key pro-inflammatory cytokine of IMIDs, upregulates arginase expression in the endothelial cells leading to impairment of nitric oxide-mediated vasodilation, and consequently to endothelial dysfunction (55–59). Furthermore, vascular endothelial growth factor (VEGF), a critical mediator of inflammation in IMIDs, is a pro-angiogenic factor that alters microvascular network contributing to the development and acceleration of atherosclerosis (60, 61). These could all contribute to higher cardiovascular risk in patients with IMIDs (54). Nevertheless, TNF-α inhibitors, which are commonly used for the treatment of IMIDs, decrease systemic inflammation and VEGF production, and thus have potential efficacy on endothelial dysfunction (56, 59, 62). Hence, the use of TNF-α inhibitors could also be a possible explanation for the lower risk of cardiovascular disease-specific mortality in patients with IMIDs.

Similar to that observed when all IMIDs were analyzed as a whole, in the analysis where each organ based IMID was separately analyzed, joint IMIDs were significantly associated with a lower risk of all-cause mortality (aHR, 0.819). Furthermore, although statistical significance was not reached, gut IMIDs (aHR, 0.930; 95% CI, 0.635–1.364) and skin IMID (aHR, 0.939; 95% CI, 0.873–1.010) showed a trend of a lower risk of all-cause mortality. Similar results were also observed in the association of organ-based IMIDs with cancer-specific mortality and cardiovascular disease-specific mortality: joint IMIDs were associated with a low risk of cancer-specific mortality (aHR, 0.512), and gut IMIDs tended to have a low risk of cancer-specific mortality (aHR, 0.730; 95% CI, 0.357–1.494). In addition, skin IMID was significantly associated with a low risk of cardiovascular disease-specific mortality (aHR, 0.790), and gut IMIDs (aHR, 0.268; 95% CI, 0.063–1.150) and joint IMIDs (aHR, 0.837; 95% CI, 0.682–1.028) tended to have a lower risk of cardiovascular disease-specific mortality. Importantly, none of the organ-based IMIDs had a higher risk of all-cause, cancer-specific, and cardiovascular disease-specific mortality.

Regarding other cause-specific mortalities, joint IMIDs were associated with a high risk of infectious disease-specific mortality (aHR, 1.397). When joint IMIDs were further subdivided, RA, but not AS, was significantly associated with a high risk of infectious-disease-specific mortality (Table 6). Among the IMIDs, RA has the widest range of biological agents with different modes of action or small molecule inhibitors available for treatment (tumor necrosis factor inhibitors, interleukin-6 receptor inhibitors, co-stimulation inhibitors, anti-B cells, and Janus kinase inhibitors) (63). Of these, interleukin-6 receptor inhibitors, used only in RA, pose a higher risk of serious infection than tumor necrosis factor inhibitors (64), commonly used throughout all IMIDs (63, 65–67). This could explain the higher risk of infectious disease-specific mortality observed only in RA.

Another interesting finding was the association between gut IMIDs and a high risk of digestive system disease-specific mortality (aHR, 5.407). Similarly, a hospital-based cohort study from South Korea also reported a high risk of digestive system disease-specific mortality in patients with IBD assessed by age-and sex-adjusted standardized mortality ratios (26). Our findings add to the previous knowledge that even after adjusting for comorbidities, digestive system disease is an important cause of death in patients with gut IMIDs. This implies the importance of optimal disease management in patients with gut IMIDs for better mortality outcomes.

This study had some limitations. First, we lacked data on the disease activity of each IMID, and we were unable to analyze whether the disease activity of each IMID was associated with the risk of mortality. In addition, we also lacked data on the duration of comorbidities and were unable to match this covariate. Second, data on alcohol consumption and smoking status are unavailable in the NHIS-NSC; thus, the possibility of confounding by these unmeasured covariates exists. In addition, data on the dose and duration of corticosteroid and immunosuppressive drugs are also unavailable in the NHIS-NSC, and we were unable to evaluate the mortality rates according to the use of these drugs. Third, we were unable to analyze psychological disease-specific mortality (i.e., suicide). However, given that the risk of all-cause mortality was lower in patients with IMIDs, we presume that psychological distress, as a cause of death, may not significantly increase overall mortality risk. Despite these limitations, using a large-scale, population-based cohort representative of the general Korean population, we were able to strictly adjust for comorbidities between patients with IMIDs and controls by conducting matching and multivariable analyses. This enabled us to investigate the impact of IMIDs, excluding the influence of comorbidities, on the risk of all-cause and cause-specific mortality.

In conclusion, IMIDs were associated with a reduced risk of all-cause mortality after adjusting for comorbidities. This was attributable to the low risks of cancer-specific and cardiovascular disease-specific mortalities, suggesting that earlier detection of cancer and cardiovascular diseases in patients with IMIDs through regular hospital visits could have led to a lower risk of all-cause mortality.

## Data availability statement

The original contributions presented in the study are included in the article/Supplementary material, further inquiries can be directed to the corresponding authors.

## Ethics statement

This study was approved by the institutional review board (IRB) of Gangnam Severance Hospital (IRB No:3-2020-0269). In addition, owing to the retrospective nature of this study, the requirement for obtaining informed consent was waived and this decision approved by the IRB of Gangnam Severance Hospital.

## Author contributions

OK, SL, JC, and KH contributed to conception and design of the study. OK, SL, JC, KH, YK, and RK organized the database. KH performed the statistical analysis. OK, SL, and JC wrote the first draft of the manuscript. OK, SL, JC, KH, YK, RK, M-CP, J-HK, YY, and HP reviewed and edited sections of the manuscript. All authors contributed to manuscript revision, read, and approved the submitted version.

## Conflict of interest

The authors declare that the research was conducted in the absence of any commercial or financial relationships that could be construed as a potential conflict of interest.

## Publisher’s note

All claims expressed in this article are solely those of the authors and do not necessarily represent those of their affiliated organizations, or those of the publisher, the editors and the reviewers. Any product that may be evaluated in this article, or claim that may be made by its manufacturer, is not guaranteed or endorsed by the publisher.
