# Supplementary material for: Risk of all-cause and cause-specific mortality associated with immune-mediated inflammatory diseases in Korea
Source: Front Med (Lausanne). 2023 Jun 20;10:1185300. doi: 10.3389/fmed.2023.1185300 (PMC10319061; doi:10.3389/fmed.2023.1185300)
Supplement: Supplementary file 1 [file Table_1.DOCX]

Supplementary Table 1. Comparison of demographics and follow-up period of patients with each IMID in our study and previous studies

|  | CD | | | UC | | | PsO | | AS | | | RA | | |
| --- | --- | --- | --- | --- | --- | --- | --- | --- | --- | --- | --- | --- | --- | --- |
|  | Present study | Ref.24 | Ref.40 | Present  study | Ref.24 | Ref.40 | Present study | Ref.41 | Present study | Ref.42 | Ref.43 | Present study | Ref.44 | Ref.45 |
| Age, years, mean | 29.07 | No data | 42.8 | 43.58 | No data | 48.59 | 46.6 | 52.19 | 41.52 | 49.3 | 48.5 | 53.86 | 58.5 | 58.0 |
| 0–19 years (%) | 0 | 0.44 | No data | 0 | 0.08 | No data | 0 | No data | 0.00 | No data | No data | 0 | No data | No data |
| 20–39 years (%) | 78.39 | 5.38 | No data | 43.07 | 3.23 | No data | 36.7 | No data | 53 | No data | No data | 16.6 | No data | No data |
| 40–59 years (%) | 15.85 | 22.05 | No data | 38.5 | 14.8 | No data | 34.78 | No data | 29.33 | No data | No data | 48.12 | No data | No data |
| 60–79 years (%) | 5.76 | 45.43 | No data | 17.4 | 42.27 | No data | 24.87 | No data | 15.67 | No data | No data | 32.51 | No data | No data |
| ≥80 years (%) | 0 | 26.7 | No data | 1.03 | 39.62 | No data | 3.66 | No data | 2 | No data | No data | 2.77 | No data | No data |
| Male sex (%) | 64.27 | 43.1 | 41.3 | 61.8 | 56.7 | 51.1 | 54.76 | 48.57 | 73.33 | 65.5 | 57.0 | 25.55 | 20.1 | 26.9 |
| Follow-up period, years, mean | 6.81 | 4.67 | 3.62 | 6.64 | 4.38 | 3.75 | 6.58 | 3.43 | 6.72 | 6.05 | 8.0 | 6.8 | 5 | 14.2 |

AS, ankylosing spondylitis; CD, Crohn’s disease; IMID, immune-mediated inflammatory disease; PsO, psoriasis; RA, rheumatoid arthritis; UC, ulcerative colitis
